# Supplementary figures and images for: Genome-wide identification and expression profiling of the COBRA-like genes reveal likely roles in stem strength in rapeseed (Brassica napus L.)
Source: PLoS One. 2021 Nov 24;16(11):e0260268. doi: 10.1371/journal.pone.0260268 (PMC8612548; doi:10.1371/journal.pone.0260268)

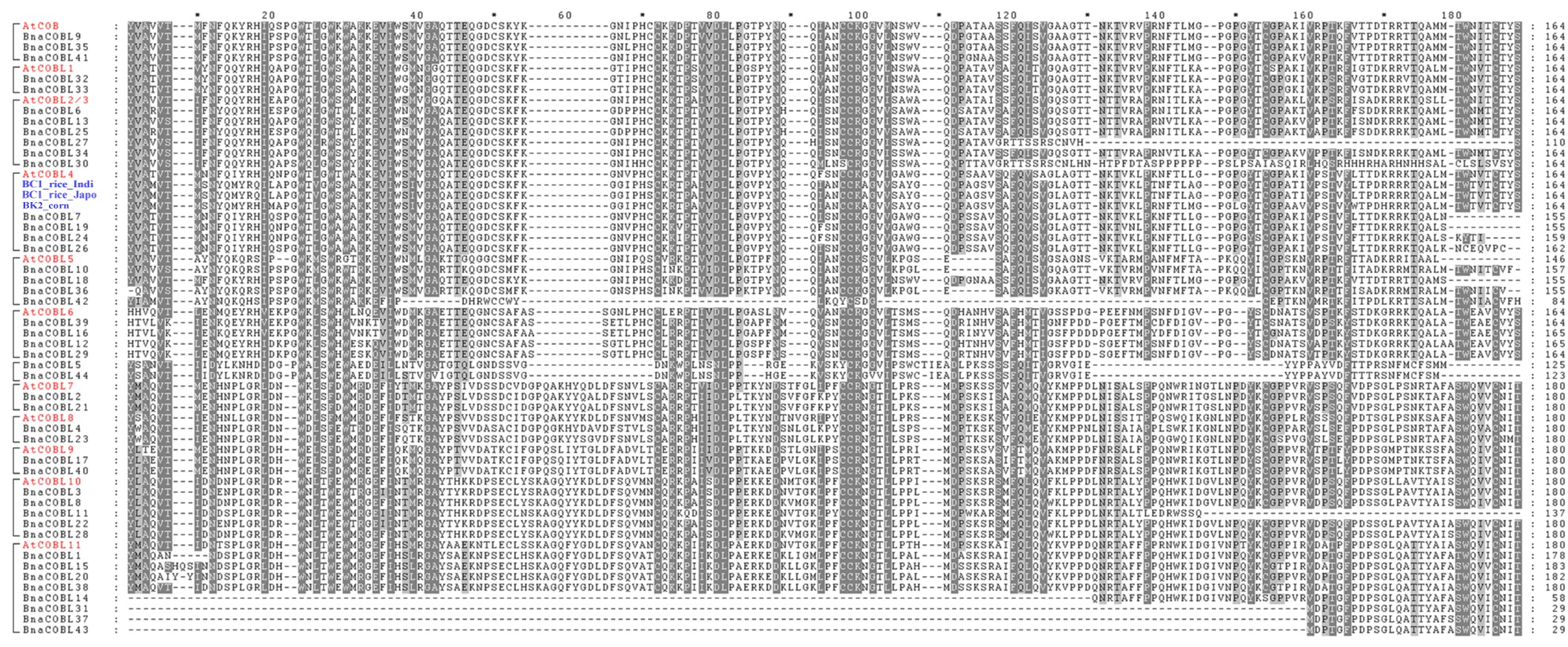

Supplement: S1 Fig — BC1_ rice_Japo (AAQ56120.1) and BC1_rice_Indi (AAQ56121.1) represent the Brittle Culm1 protein in Oryza sativa subsp. Indica and Oryza sativa subsp. Japonica respectively. BK2 (ABJ99754.1) encodes brittle_stalk-2 protein in corn. (TIF) [file pone.0260268.s001.tif]
