# Supplementary material for: Genome-wide identification and expression profiling of the COBRA-like genes reveal likely roles in stem strength in rapeseed (Brassica napus L.)
Source: PLoS One. 2021 Nov 24;16(11):e0260268. doi: 10.1371/journal.pone.0260268 (PMC8612548; doi:10.1371/journal.pone.0260268)
Supplement: Attachment — Submitted filename: WHQ comments.docx [file pone.0260268.s008.docx]

In this paper, using the method of biological information, 44 *COBRA*-like genes were identified in rapeseed. Then, the phylogenetic relationships, the chromosome location, the domain composition, and the putative cis element distribution of *BnaCOBLs* were determined. Different expression levels in stems with distinct lodging resistance suggested that there was some association between *BnaCOBLs* and stem lodging resistance. The logic of the article is very clear, and the scientific issues of concern are also well explained. But there are still several issues that need attention:

(1) In line 127, when determining the promoter, you should not only use the length as the standard, but should combine some basic characteristics of the promoter, such as the -10 region and the -35 region. Because the promoter may exist in the last gene, or the two genes mentioned in your article share the promoter. The sequence of the promoter directly affects the results of the subsequent analysis. For example, the speculation of gene function in line 445 may be biased.
(2) Line 117. Why you choosed 10 motifs and motif width of 8-50 amino-acid residues as this papaer's parameters. The basis should be pointed out here.
(3) In the discussion section, when the article directly quoted the content of the article results, the corresponding picture number was not marked. I think it will be easier for readers to read this article by marking the corresponding picture numbers.
