# Supplementary material for: Genome-wide identification and expression profiling of the COBRA-like genes reveal likely roles in stem strength in rapeseed (Brassica napus L.)
Source: PLoS One. 2021 Nov 24;16(11):e0260268. doi: 10.1371/journal.pone.0260268 (PMC8612548; doi:10.1371/journal.pone.0260268)
Supplement: Attachment — Submitted filename: Responses to the Refrees Comments.docx [file pone.0260268.s009.docx]

**Dear Referees:**

We thank the reviewers for their enthusiasm and interest in this research. We would like to acknowledge the referees for spending their time and effort sharing their views and providing constructive comments. These comments help us improve our manuscript. The detailed response is provided below following the referee’s specific comments.

**Responses to the Referees’ Comments**

**Reviewer #1**

In this manuscript, the authors conducted a genome-wide analysis of the *COBRA-like* gene family in rapeseed. They also performed phylogenetic analysis, cis-element, and gene expression analysis. However, I do have some concerns before its final acceptance.

**1**. In phylogenetic analysis COBLs, authors had done analysis using protein sequences from *Brassica* and *A. thaliana* only. They can draw a better picture/results if they use more species for phylogenetic analysis.
**Response**

As the referee suggested, we can use more species for phylogenetic analysis to draw a better picture. However, here we aimed to reveal the expansion of *BnaCOBLs* family, and therefore we chose only both diploid progenitor species *B. rapa* and *B. oleracea* for phylogenetic analysis and used *A.thaliana* as an outgroup.

**2**. Why authors randomly selected the five genes out of 44 for Q-RT PCR analysis? In my opinion, there should be a criterion/logic for selection. Like, (a) selection based on their subclass division; phylogenetic based, (b) selection of genes based on their in-silico expression results.
**Response**

Based on the phylogenetic subgrouping and *in silico* expression patterns of *BnaCOBLs*, we selected the five genes for qRT-PCR analysis. Among these genes, *BnaCOBL9*, *BnaCOBL35*, and *BnaCOBL41* belong to the subgroup AtCOB while *BnaCOBL19* and *BnaCOBL24* to AtCOBL4.

3. “*BnaCOBLs* were involved in stem lodging resistance” based on these experiments, it overestimated results. In my opinion, some more experiments should be conducted to further functional validations of results to meet the journal standards. Functional validation of genes using transgenic approach/VIGS/CRISPAR.
**Response**

Thank you for your suggestion. Our results provide some informative clues for stem responses of defense to abiotic and biotic stress. Next we will carry out transgenic research and gene editing to validate whether these genes are associated with stem lodging in *B. napus*.

4. It is also suggested that authors should improve the discussion section. They have to discuss their results and compare them with some earlier and recently published papers in more depth and clarity.
**Response**

As the referee proposed, we have carefully revised the Discussion section in this revision. After analysis of the protein sequence of *COBLs*, we further discussed the structural character of *COBLs* in rapeseed. The expression profile was refreshed, too.

5. Please provide the Pfam ID used for COBL gene identification.
**Response**

The Pfam ID of the *COBRA-like* gene family is PF04833. We added up this Pfam ID to the revised manuscript.

6. Detail of BnaActin gene used in this study. i.e., NCBI ID, or any other database ID should be provided.
**Response**

As listed in S1 Table, we used *BnaActin* (*BnaA10g06670D*) to normalize the expression levels of mRNAs.

7. How many biological replicates of each tissue were used for qRT-PCR analysis, since it is not clear from the martial and methods section. Please clear?
**Response**

Thank you for your reminding us. Three biological replicates were used for qRT-PCR analysis, which is shown in lines 193-194 of the revised manuscript.

8. How they named COBL genes? Please provide details.
**Response**

*COBLs* is the abbreviation of *COBRA-like* genes. The number suffixes were added according to the chromosome location from chromosome A01 to chromosome C09. The details for the naming of *BnaCOBLs* were illustrated in the legends of Figure 2.

**Reviewer #2**

In this paper, using the method of biological information, 44 COBRA-like genes were identified in rapeseed. Then, the phylogenetic relationships, the chromosome location, the domain composition, and the putative cis-element distribution of BnaCOBLs were determined. Different expression levels in stems with distinct lodging resistance suggested that there was some association between BnaCOBLs and stem lodging resistance. The logic of the article is very clear, and the scientific issues of concern are also well explained. But there are still several issues that need attention :

**1**. In line 127, when determining the promoter, you should not only use the length as the standard, but should combine some **basic characteristics of the promoter**, such as **the -10 region and the -35 region**. Because the promoter may exist in the last gene, or the two genes mentioned in your article share the promoter. The sequence of the promoter directly affects the results of the subsequent analysis. For example, the speculation of gene function in line 445 may be biased.
**Response**

As the referee suggested, we reviewed the literature on *cis*-acting regulatory elements (CAREs) research once again. Although the CAREs of one gene could locate at the upstream, downstream, and introns of this gene, we only took the CAREs in the upstream promoter regions into account in our research. First, limited to the lack of 5’ untranslated region annotation, the start codon was selected as the ending site of promoters. Then, referring to researches on other gene families, the *cis*-elements within 1500bp upstream of the start codon on both strands were identified by screening on the online database PlantCARE. At last, we checked the TATA- and the CAAT-box in the proximal region (-500bp) on the same strand of the gene, and remained all other *cis*-acting regulatory elements on both strands. Because the core promoter regions are direction-sensitive, but the elements in the distal region are direction-insensitive. Two *BnaCOBLs* were found to lack of TATA-box. But this phenomenon seemed to be common in *B.napus* referring to that the TATA-less promoters account for 79.9% of all promoters in *Arabidopsis.* So all the promoters are used for the subsequent analyzes. The corresponding methods and results were re-written in the revised manuscript, so did S5_Table and Fig_5 .

2. Line 117. **Why** you choosed 10 motifs and motif width of 8-50 amino-acid residues as this papaer's parameters. The basis should be pointed out here.
**Response**

We made a mistake because this part of methods should not be present in our manuscript. Once, we had analyzed protein structure by MEME which could only provide unknown motifs. Then we insteadly used the known domains of COBL family to evaluate *BnaCOBLs*. These results could display which functional domains have been differentiated among subgroups of *BnaCOBLs.* Furthermore, we added multi-sequence alignments figures (S1_fig and S2_fig) to illustrate the amino acid differences.

3 In the discussion section, when the article directly quoted the content of the article results, the corresponding picture number was not marked. I think it will be easier for readers to read this article by marking the corresponding picture numbers.
**Response**

Thank you for your advice. We listed corresponding figures and tables when quoting the results in the Discussion section of the revised manuscript.
