# Supplementary material for: Genome-wide identification and expression profiling of the COBRA-like genes reveal likely roles in stem strength in rapeseed (Brassica napus L.)
Source: PLoS One. 2021 Nov 24;16(11):e0260268. doi: 10.1371/journal.pone.0260268 (PMC8612548; doi:10.1371/journal.pone.0260268)
Supplement: Attachment — Submitted filename: Response to Reviewers.docx [file pone.0260268.s010.docx]

**Dear Referees:**

We thank the reviewers for their enthusiasm and interest in this research. We would like to acknowledge the referees for spending their time and effort sharing their views and providing constructive comments. These comments help us improve our manuscript. The detailed response is provided below following the referee’s specific comments.

Reviewer #3: Yang et al., used bioinformatics method to analyze the COBL gene family in rapeseed. The results are interesting, and may have potential guiding significance for lodging resistance improvement. The major comments are:

1. Whether the animal (like mouse or human) has COBL homeologues, if has, please introduce the gene function of COBL genes in Introduction.
The COBRA-like gene family (pfam04833) is the only member of the superfamily cl04787 which only be found in embryophyte from a search of the conserved domain database (Search on 1st Sep, 2021).

2. The authors are better to give the uniform naming in B. napus, B. rapa and B. oleracea based on Fig. 1 result, and combine the Table S2 and S3 together. Otherwise the readers hard to compare the same homeologue in different species.
As you recommended, we have renamed the *COBL* genes of *B. rapa* (20) and *B. oleracea* (23) as *BraCOBL1-20* and *BolCOBL1-23* based on their chromosome locations. We combined the Tables S2 and S3 together to form a new S2 Table.

3. Based on the protein location predication result, the BnaCOBL10 and BnaCOBL42 were not on cell membrane, while these two genes were in the same subgroup (Fig. 2). The authors may discuss the possibility of the differentiation of gene function in Discussion.
Both *BnaCOBL10* and *BnaCOBL42* are orthologous from *AtCOBL5*, that is the only member without the GPI*ω*-attachment site in *Arabidpsis*. The GPI*ω*-attachment site is important for COBL proteins to be anchored in plasma membrane. So the orthologs of AtCOBL5 subgroup in rapeseed are not predicted to be on the plasma membrane. The function of the gene *AtCOBL5* in Arabidpsis is little known, so did the orthologs of AtCOBL5 subgroup in other plants.

4. The authors need to check the italics of the gene name fully (e.g. Line 37, 44, 56, 57, 79, 141, 223, 226, 229, 230 etc.)

The words with capital letters in lines listed by reviewer, are not the names of genes but the names of subgroups or gene families. It is not necessary to italic the names of subgroups or gene families.

Reviewer #4: In this manuscript, 44 COBRA-like genes were identified in rapeseed by sequence identity and conservative COBRA domain. The author characterized these genes on phylogenetic grouping, the chromosome location, the domains, and the putative cis-element distribution in promoters of BnaCOBLs. The expression profiles and the protein domains of AtCOB and AtCOBL4 subgroup imply these AtCOB subgroup members may be involved in stem development and stem breaking resistance of rapeseed, rather than members in AtCOBL4 subgroup, which are functional in crops of grass family. The logic of the article is clear, and the scientific issues of concern are also well explained. But I think authors should address following points in this manuscript before publishing in “PLOS ONE ”:

1. The conclusion in line 79 to 80 seemed to be expanded according to the results and discusses.
Based on our results we redrawn the conclusion.

2. As the topology of the phylogenetic tree (Fig 1), BnaCOBL5 and BnaCOBL44 did not belong to any subgroup orthologs from AtCOBLs. Where they evolved from? Maybe these two genes are not the members of COBL family in rapeseed.
The *BnaCOBL5* and *BnaCOBL44* contain the COBRA domain, which meets the criteria of a member of COBL family. In fact, analysis of identity indicated both genes evolve from *AtCOBL2/3* orthologs.

3. The alternative splicing of subgroup AtCOBL4 presented in the Result section should be supported by figures.
As shown in Fig4.tif, we illustrated the skipped exons in rectangle with dotted line.

4. Why the members in AtCOBL4 subgroup with incomplete protein still are expressed in stems at a certain level?
This phenomenon is caused by the unchanged promoters of members in AtCOBL4 subgroup.

5. In line 19 in the Abstract section, whether the expansion of COBL family in B. napus was attributable to ‘whole-genome duplication’ or ‘whole-genome triplication’ as written in line 423?
It is ‘whole-genome triplication’ in line 19.

6. Some syntax and formatting errors such as, in line 243, ‘The kind of gene pair was’ should revise as ‘This kind of gene pairs were’ in this context; in Table 1, the ‘p-value’ in line where the ‘Gene Name’ is ‘BnaCOBL20’, does not line up with other elements in the same line.
We carefully checked and corrected the grammatical and formatting errors in the revised manuscript.

7. Add some correlation descriptions between the genes (such as BnaCOBL9, BnaCOBL35 and BnaCOBL24 etc.) expression change and stem strength in results.

We have described the expression change patterns in line 376 to 382.
